# Prediction of Seizure Recurrence. A Note of Caution

**DOI:** 10.3389/fneur.2021.675728

**Published:** 2021-05-13

**Authors:** William J. Bosl, Alan Leviton, Tobias Loddenkemper

**Affiliations:** ^1^Boston Children's Hospital, Boston, MA, United States; ^2^Harvard Medical School, Boston, MA, United States; ^3^Health Informatics Program, University of San Francisco, San Francisco, CA, United States

**Keywords:** electroencephalography, machine learning, chaos & non-linearity, dynamical systems, seizure prediction

## Abstract

Great strides have been made recently in documenting that machine-learning programs can predict seizure occurrence in people who have epilepsy. Along with this progress have come claims that appear to us to be a bit premature. We anticipate that many people will benefit from seizure prediction. We also doubt that all will benefit. Although machine learning is a useful tool for aiding discovery, we believe that the greatest progress will come from deeper understanding of seizures, epilepsy, and the EEG features that enable seizure prediction. In this essay, we lay out reasons for optimism and skepticism.

## Introduction

In a 2016 Epilepsy Foundation research program survey of people with epilepsy, “unpredictability was selected as a top issue regardless of seizure frequency or severity” (1). Seizure recurrence can be severely limiting (e.g., no driving), (2) socially disruptive and stigmatizing (3), and even life-threatening (4). Consequently, seizure prediction has the potential to improve epilepsy management and, therefore, the quality of life of persons with epilepsy (5).

Successful methods to predict an imminent seizure based on electrographic signatures, and potentially intervene with, for example, “responsive neurostimulation” (RNS) (6–8), would allow alternatives to drugs to minimize seizure recurrence. When a “pro-ictal” (9, 10) or “pre-ictal state” (11, 12) is identified, this mode of therapy provides highly localized stimulation intended to interrupt a seizure. Identification of times of greater and lesser seizure susceptibility will likely benefit patients, as such states may permit urgent care and interventions.

Progress in our understanding of how seizures develop and propagate (13) would lead to the expectation that “it may be possible to provide seizure prediction to a wider range of patients than previously thought” (14). We are not so sure. Before we explain why we expect only limited success anytime soon, we briefly review what is new with seizure prediction and potential electronic interventions for refractory epilepsy.

Clinicians and patients have long known that some seizures can be preceded by warning signs or symptoms (15). By and large, only about a quarter of patients with generalized epilepsy acknowledge an aura (15).

Electrocorticography (ECoG), which records from electrodes placed directly on the exposed surface of the brain, appears to be the best way to gather all the information for surgical removal of a seizure focus (16). ECoG signals are physically identical to EEG signals. Since they are placed directly on the cortex, less noise contaminates the signal and electrodes can be more closely spaced. Otherwise, the raw data from ECoG that might be fed into a machine learning algorithm is the same as with EEG data. The prediction of seizure recurrence now seems possible with scalp electrodes (17–31). Nevertheless, “modest outcomes associated with localization of abnormal electrophysiology suggest … a fundamental gap in our understanding of how neurophysiologic biomarkers relate to pathophysiology” (32).

Seizure prediction algorithms can be characterized in a variety of ways. One perspective is to consider three independent aspects or axes: (1) the physiological signal to be measured, such as brain electrical activity or heart rate; (2) signal processing methods, which compute various signal features; and (3) machine learning methods, which take signal features as input and attempt to find patterns of features that distinguish seizure activity from non-seizure activity. Advances in signal features and machine learning algorithms continue to advance rapidly and contribute to improved seizure prediction. Signal features that are associated with seizures appear to be patient-specific. Hence, to have any value, machine learning algorithms need signal features from many seizures over a long period of time (33). EEG measures of brain electrical activity continue to be the most common physiological measure associated with seizures, but other measures based on cardiac function, dermal response, or movement are also used (34).

## Background for Understanding Seizure Generation, Inhibition, and Propagation

In the next few paragraphs, we review some elements of seizure generation and propagation that might aid in understanding electrographic correlates of seizures.

### Epileptogenic Zone

The epileptogenic zone (EZ) is tautologically defined as the brain area indispensable for seizure generation (35). Among patients with focal epilepsy, more than 90% of seizures have discharges in the seizure focus and not elsewhere (36). In some patients, however, complete resection of the presumed EZ did not lead to seizure-freedom (37). Post-surgical recordings of these patients suggest that areas adjacent to the resection were also triggering the epileptic seizures. So was born the concept of “potential seizure-onset zones” (37).

Deeper gray structures (such as the thalamic reticular nucleus) appear to modulate the onset and propagation of other seizure phenomena (e.g., epileptic 2–4 Hz spike-wave discharges) (38). In addition, the epileptogenic zone in patients with pharmaco-resistant seizures can be larger than in people whose seizures are more readily controlled with medication (39).

### Seizure Propagation and Networks

Seizure generation is only the beginning. Seizures are propagated **“**when synchronous connected groups of neurons work in tandem with rapidly changing de-synchronous relationships from the surrounding epileptic network” (40). The balance between inhibition and propagation, and—to a certain extent—underlying structural and functional connectivity, will determine to what extent the seizure does or does not spread (41). One seizure onset pattern is characterized by hypersynchrony and progressive impairment of inhibition leading to seizure propagation (42).

Seizures are currently defined by the area and signal recorded. As identification of these improve, so will seizure definition and seizure detection. Examples are intensive care patients who had a much higher percentage of seizures detected by intracortical depth electrodes than by surface EEG (43). Therefore, higher spatial resolution, and evaluation of additional signal characteristics have the potential to influence our perception of seizures. Hence, seizure prediction hinges on our definition of seizure onset, which is likely to change as detection techniques improve.

Inter-neuronal activity in the cortex can restrain the spread of epileptiform activity (44). As might be expected, seizure propagation is enhanced when local inhibition networks are defective (45).

Although many reports of brain functional connectivity have assumed “temporal stationarity” (i.e., no change with time), brain networks do reorganize almost continuously in response to both internal and external stimuli, resulting in temporal fluctuations of functional connectivity within and between networks across multiple time-scales (46, 47). By “coordinating excitability between brain regions in the epileptic network,” changes in functional connectivity between/among networks not only allow propagation of the seizure activity, but might “enhance initiation, evolution, and termination of seizures” (32). The widespread disturbances of structural and functional connectivity that characterize some seizure disorders also appear to contribute to treatment resistance (48).

Epilepsy is considered to be a disorder of neural network organization (49). Research in network science has shown that small changes in network structure can have very large effects on network function, just as small changes in initial conditions can have large effects on time series (50). This suggests that small changes to a non-epileptic neural network may be all that's needed to make the brain epileptic. Similarly, small changes in just the right brain regions may be all that's needed to reduce seizures. Although this has not yet been demonstrated in humans, tools for measuring functional cortical networks are now available (51).

The signal variability of local connectivity among people with epilepsy appears to be significantly higher than in healthy controls (52), bringing excitability of the cortical neurons more often closer to the tipping point of seizures. Although network connectivity in seizure-onset zones can be increased during inter-ictal epochs (32, 53), ictal electrographic patterns appear to be generated by network mechanisms that are different from those sustaining inter-ictal potentials (54). Even brief focal spikes can activate diffuse distant networks (55), supporting the characterization of epilepsy as a network disease (56, 57).

### Electrographic Correlates/Patterns /Signatures of Seizures

In one third of patients with a diagnosis of pharmaco-resistant focal epilepsy who are candidates for therapeutic surgery, fast activity at 80–120 Hz associated with very slow transient polarizing shift, and voltage depression appear to be excellent biomarkers of ictogenesis and reliable indicators of epileptogenic zone boundaries (58). The high rate of co-occurrence probably reflects the restrictive criteria used to select candidates for surgery who have a presumed single-seizure-focus. Others have found spectral power in discreet frequency bands, as well as time- and/or frequency-domain inter-channel correlations to be helpful (14, 59).

Still other seizure onset patterns are characterized by de-synchronization of background activity and the appearance of fast low-voltage rhythms (41, 42), while excessive synchronization correlates with termination of the seizure (60). The seizure evolution pathway appears to differ among patients and tends to be stereotypical for each individual (11, 13, 61). Consequently, for prediction purposes, ictal electrographic signatures need to be individualized for each person for each seizure type (5, 14, 17, 20, 23, 25, 31, 56, 62–72). The buzzword is “patient-specific.” Perhaps “big data” should be another buzz-word because analyses of large sample sizes and multiple individual variables will be needed to decide if groups of patients with similar epilepsy types and other physiological or demographic conditions can be viewed as a (relatively homogeneous) group.

### Chaos and Chaotic Systems

Unlike its meaning in common parlance, “chaos” does not mean random, but only practically unpredictable. Even though the current state of the system might be known almost infinitely precisely, the smallest error or perturbation limits our ability to predict future states of the system.

Seizures often appear to be surprising. This apparent unpredictability might reflect purely random phenomena, or emergent chaotic phenomena that can arise at any time. If seizures are random, then prediction may be impossible in most cases until the pre-seizure changes begin to occur. If seizures are emergent chaotic phenomena, seizure prediction should be possible, since chaotic systems are deterministic. However, the chaotic nature of the system may limit the pre-seizure prediction time.

*Non-linear (or chaotic)* systems are composed of parts that can interact in complex ways, even if the parts themselves have simple dynamics or behavior. Non-linear systems are characterized by sensitive dependence on initial conditions, emergent phenomena, spontaneous order or synchronization between components, adaptation, and feedback loops (defined below), all of which result from the complex interaction of the parts. The EEG patterns of epilepsy appear to be non-linear (73, 74), likely reflecting non-linear dynamics of the brain.

*Emergence* has been defined as “the arising of novel and coherent structures, patterns and properties during the process of self-organization in complex systems" (75). This process of “self-organization” consists of adaptive behaviors between parts that emerge within chaotic systems, leading to a limited number of relatively stable configurations (76). The non-epileptic brain is stable and does not easily move into an ictal (seizure) state. It exhibits a property called “dynamical resistance” to seizures, which refers to a resistance to transitions to a seizure state (77). Resilience, a similar dynamical property, describes a system's ability to maintain normal function when internal errors or external environmental conditions arise (78). The epileptic brain may have reduced dynamical resistance and/or resilience, resulting in “multistable dynamics,” (79) which means that it may spontaneously self-organize into a stable ictal state (80, 81). Dynamic networks based on EEG channel synchrony or coherence (amplitude synchrony) of the EEG may also differentiate patients with generalized epilepsy from normal controls (82).

*Sensitive dependence on initial conditions* is exemplified by the butterfly effect. In the highly non-linear atmospheric system, a small perturbation produced by a butterfly can lead to large changes at a future time, perhaps even a hurricane. In short, an arbitrarily small change in the state of a non-linear system at one time can have a large effect later. This is what makes a deterministic non-linear system practically unpredictable. It is not yet known if seizure occurrence (as opposed to the underlying neural spiking activity) follows a deterministic, chaotic pattern, or if it is simply a purely random process (83, 84).

Nobody is in charge of food distribution for most major cities and yet food gets distributed. This characteristic of complex systems is identified as spontaneous order, which may represent what occurs during the inter-ictal resting state (85). Another perspective is that the ictal and inter-ictal states each represent a stable, or semi-stable, attractor state of the dynamical system. An epileptic brain transitions between these states relatively easily, while this phase shift is very difficult to induce in a non-epileptic brain.

Neural connectivity, information transmission, and processing that are essential functions of the brain, may be altered on a large scale to allow the brain to switch into pathological states such as seizures, suggesting a scale dependent tipping (critical) point between normal physiologic function and pathological spread of electrical activity (86). However, if the neural structure of the brain is near a critical point, small changes in neural network structure may tip the brain into an unstable regime where seizures can occur spontaneously. This kind of spatial sensitivity to small changes has been described for networks (87).

### Pre-ictal

If seizure prediction is to become clinically useful, programs that analyze electrical activity need to identify the pre-ictal state as early and reliably as possible before seizure onset. At present, we do not know when the pre-ictal state begins. Knowing when the pre-ictal state begins will allow an assessment of the time needed to detect and interrupt an impending seizure.

Dynamic models of events define different phase transitions (some with and others without an event or characteristic) and then model the probability of transitions from one state to another (88–91). People who work on seizure-prediction algorithms recognize at least three states: a seizure (ictal) state, a pre- or pro-ictal state, and all others. Machine-learning programs are given the task of comparing the electrographic characteristics of variously defined time intervals before a seizure to the electrographic characteristics of times further away (in time) from seizure onset. The goal is to define a pre-ictal state. To do this effectively, the machine-learning programs need to be provided an abundance of EEG recordings (92), which are becoming increasingly available.

### Characteristics of Ictal EEG

One group found that a few hours before a seizure, the “network states become less variable (“degenerate”), and this phase is followed by a global functional connectivity reduction” (93). Others have reported “less chaos” (94, 95) or “increased synchronization” before a seizure (18, 96). One group found that prior to seizure onset, the amplitude of pre-ictal discharges progressively increased as the interval between these discharges gradually decreased (97), while others have found that the cumulative energy profile (98), or measures of spectral entropy, spectral energy, and signal energy can help identify pre-ictal states (17). Still others have emphasized that the best discriminators vary for each individual (99), while another group emphasized the co-occurrence of multiple phenomena in a high potassium hippocampal slice model (loss of neuronal network resilience within the setting of critical slowing down, decreased ability of a network to recover from perturbations, increased high frequency fast activity, and successively decreasing resilience to stimulation (100).

### Timing

The goal is to be able to identify the increased seizure propensity sufficiently before the seizure onset. The interval between identification of the likelihood of an impending seizure and the occurrence of the seizure has varied considerably, from under 10 s (17, 23, 24, 72, 81, 101, 102) to intervals of an hour or more (20–22, 36, 93, 103).

### Periodicity

Seizures can display multiple types of periodicities (e.g., circadian, multi-day, weekly) in dogs (104) and humans (5, 105–109). Because only some people have seizures that occur with an obvious periodicity, seizure prediction is best viewed as patient-specific (5). Changes in level of epileptogenicity (state transitions) (110, 111) that most likely characterize periodicities are best viewed as contributing information to seizure-forecasts (112). To what extent these periodicities reflect changes in high-frequency oscillations (112), EEG spike potentials (112), brain connectivity, and inhibitory neurons (113) remains to be quantified. Seizure prediction algorithms are most likely to be effective when they include all the variables that provide relevant discriminating information for that patient. Each individual's seizure periodicities, once quantified, may be among those discriminating information.

### Warning Signals Before Critical Transitions

The existence of early warning signals before catastrophes (e.g., species extinction, pandemics) (114–117) supports the concept that gradual transitions from stable to unstable conditions can reach a tipping point that heralds the irreversibility of the transition (118). Phase transitions in chaotic systems can happen either gradually or suddenly, depending on the system (119). Indeed, the relatively early aspects of the transition from a non-seizure state to seizure activity can be gradual (54, 120, 121) and widespread (36). “The suitability of typically applied early warning indicators for identifying heightened probability of a seizure remains controversial” (122, 123).

### Binary Forecast or Probability Estimate of Seizure Risk

The seizure detection system can provide a binary forecast (impending seizure: yes/no), or a forecast that provides an estimated probability of an impending seizure (91, 124). The probability forecast, though obviously more informative than a binary forecast, will likely be degraded to a binary forecast when algorithms are written to initiate responsive neurostimulation (8). Even binary forecasting systems (high- and low-risk), using only patient-reported seizure data, correctly predicted seizures in about half of 50 patients (125).

### Relatively Reliable Prediction

In an international crowdsourcing competition, an appreciable number of the more than 10,000 algorithms submitted by 478 teams were able “to distinguish between 10-min inter-seizure versus pre-seizure data clips” for each of three patients based on 442 days of continuous intracranial electroencephalography recordings from 16 subdural electrodes (14). These results prompted the authors to conclude, “clinically-relevant seizure prediction is possible in a wider range of patients than previously thought possible.” While these results are promising, they are limited to three patients. As noted previously, different patients, or different epilepsy types, may have different pre-ictal time periods, ranging from seconds to an hour or more. Much larger patient sample populations will be needed to map out the limits of pre-seizure prediction. As a first step in this direction, crowdsourcing analysis of intracranial EEGs continues on related platforms, such as epilepsyecosystem.org (14, 126).

### Not so Reliable Prediction

Despite subsequent expressions of enthusiasm (12, 31, 92, 127, 128), others have found that the EEGs of one third of patients with focal (129) or multifocal (130) epilepsies were not able to provide adequate predictive information about impending seizures. Findings such as these prompt us to offer words of caution about the anticipated capability to predict seizures and intervene effectively to prevent seizure occurrence.

In our acknowledging that some, perhaps many, people with seizures will benefit from machine-learning programs that predict seizure recurrence, we also want to justify the restraint in our enthusiasm. We do so based on the following considerations.

### Prediction Performance Metrics

Specification of system parameters, such as prediction period, prediction horizon and data-driven characterization of lead seizures (minimal duration of seizure-free period) each influence prediction performance metrics (131). Consequently, investigators have the opportunity to cherry-pick the system parameters that will variably maximize their metrics. To minimize this, one group proposed a test metric of the difference between algorithm sensitivity and chance sensitivity given an equal proportion of time spent under warning (132).

Prediction performance metrics may include indicators of sensitivity and specificity (59). Sensitivity is defined as the total number of seizures being accurately predicted divided by the total number of seizures recorded. Specificity is the number of correctly-identified non-events and is usually more difficult to evaluate due to the relatively small number of seizure events during most time intervals (133). Performance indices related to specificity include time in warning (the fraction of time the system makes positive predictions), and false positive error rate (8, 132).

A more general measure of performance that summarizes the tradeoff between sensitivity and specificity is the area under the receiver operating characteristic (ROC) curve (AUC) that discriminates between inter-ictal and pre-ictal data and is the preferred measure for many studies benchmarking multiple seizure forecasting algorithms (59, 132, 134).

The ROC curve is a plot of True Positive Rate (TPR) or sensitivity, vs. the False Positive Rate (FPR), or 1-specificity for varying model parameters. Thus, the area under the ROC curve (AUC) is a measure that accounts for the relative trade-off between sensitivity and specificity. Both are needed for a prediction algorithm to be practical. For example, perfect sensitivity is always possible if specificity is completely sacrificed: always predict a seizure and every seizure will be correctly predicted, 100% of the time. Similarly, always predicting “no seizure” will never falsely predict a seizure and thus have perfect specificity. Clearly, neither of these extremes is useful. Optimizing both sensitivity (predict all seizures) and specificity (no false alarms) is the ideal. The AUC is a measure of this optimal balance (135).

Because of the potential problem of overfitting (136) of the evaluation statistical model, investigators now seek to measure an “optimism corrected AUC,” which corrects for/avoids optimism by either: cross-validation with replication (137–139) or leave-pair-out cross-validation (140).

Variations or extensions of this theme include a final “Improvement over Chance” binary metric that compares the measured AUC to a “chance-level AUC” (141), accuracy rates based on ROC curves (142), and an ROC analysis to extrapolate a cut-off value for the most significant predictors of seizure recurrence (143).

Another potential approach to assessing the accuracy of a prediction algorithm is to compare its accuracy to that obtained using surrogate output data that has some of the properties of the true data. An example of this is to randomly permute or shuffle the outcomes labels, thus retaining the same number of positives and negatives as in the original outcomes. (144). After permuting the labels, the predictive accuracy, including sensitivity and specificity, is computed. This process is repeated many times in a Monte Carlo style simulation, and the accuracies for all of the surrogate trials are accumulated to determine how likely the predictive accuracy with the true labels can be attained by random chance. For reasons that are not clear, this type of Monte Carlo simulation, which can be used to estimate the probability of attaining a selected AUC (145) has been used less frequently (146–148).

## Discussion

### Butterfly Effect/Important Data Missing

The butterfly effect refers to the sensitive dependence of a non-linear system on the accuracy of measurements at a given starting time. Prediction of the future state of a non-linear system is limited by the butterfly effect. For example, even if all of the exact physical equations for atmospheric dynamics are known, predicting the weather more than a few days into the future is limited by how accurately the present weather conditions can be measured at every location from the surface of the earth to the top of the lower atmosphere. If neural function is a non-linear system, then seizure prediction may be limited by the butterfly effect.

The butterfly effect results from the slightest measurement imprecision. This is different from a lack of information about all the important processes involved in seizure generation or a lack of data. We prefer to use the word “missingness” (149, 150) to describe a lack of measured data regarding the processes involved in seizure onset and spread.

Seizure prediction will enable successful intervention only if identifiable pre-ictal signatures occur sufficiently clearly and sufficiently early to enable a predictive model to be constructed. A few reviews of the many applications of signal processing and predictive algorithms present the enormous breadth of this effort (151–154). This approach has begun to be applied to seizure prediction (155, 156) with the recognition that the amount of raw EEG data needed for deep learning approaches might be prohibitively large (157).

### One Size Does Not Fit All

An algorithm created for one person is unlikely to predict seizure recurrence in another (5, 11, 13, 14, 17, 20, 23, 25, 31, 56, 61–72). Another potential problem is that although seizure prediction is specific to an epilepsy or seizure type, prediction can be conditioned by myriad patient characteristics. Large amounts of patient data, together with properly used machine learning algorithms, are likely needed to identify the best way to apply seizure prediction for optimal patient benefit. Sufficient amounts of data from many patients may improve the ability of patient-independent algorithms for the benefit of patients and their physicians who would strongly prefer not to have to wait a year to receive benefits from the prediction capability of wearable devices. However, it is also clear that seizure prediction algorithms can learn from patient-specific patterns and improve over time scales from days to months (158–160).

Much of the success of the seizure prediction field is owed to those investigators who have created a valuable database, made it publicly available, and asked others to contribute to this culture of data sharing (161). Many annotated seizure databases exist. Some of the better known ones can be explored further in these references: (71, 125, 161–165).

Research using machine learning algorithms is frequently hampered because of the lack of standards that allow data from disparate databases to be aggregated (166). A lack of sufficient amounts of publicly-available data is also apparent (154). Because insufficient available human data were (59), a recent Kaggle machine learning competition for seizure prediction relied on canine EEG data. Advances in seizure prediction will be enhanced if the epilepsy research community can collaborate to create a common, aggregated, publicly-available data resource as the genomics community has done for the Human Genome Project (167).

We are cautiously optimistic that many people will benefit from an ability to predict seizure recurrence. We do, however, want to temper optimism that this ability will be available to nearly all patients and all seizures. Very large seizure data sets, with proper clinical annotation, and machine learning algorithms, as well as deeper understanding of the dynamics and neurophysiology of seizures and epilepsy, will be needed to provide a much clearer picture of the limits and possibilities of seizure prediction.

## Data Availability Statement

The original contributions presented in the study are included in the article/supplementary material, further inquiries can be directed to the corresponding author/s.

## Author Contributions

AL and TL conceived of the original idea for this paper. AL wrote the first draft. WB wrote substantial sections on non-linear systems, chaos theory, and EEG processing. All authors contributed to the article and approved the submitted version.

## Conflict of Interest

TL is part of patent applications to detect and predict clinical outcomes, and to detect, manage, diagnose, and treat neurological conditions, epilepsy, and seizures. TL is co-inventor of the TriVox Health technology and Boston Children's Hospital might receive financial benefits from this technology in the form of compensation in the future. WB is named along with TL on a patent for epileptogenicity that is owned by Boston Children's Hospital. The remaining author declares that the research was conducted in the absence of any commercial or financial relationships that could be construed as a potential conflict of interest.
